# One-lung ventilation and 3D image analysis in a case of tracheal bronchus with steeply angled branching of left main bronchus: a case report

**DOI:** 10.1186/s40981-022-00545-0

**Published:** 2022-07-25

**Authors:** Taichi Onimaru, Mineto Kamata, Hideyuki Nakagawa

**Affiliations:** 1 Department of Anesthesiology, Saitama Medical University International Medical Center, Saitama, Japan; 2 Department of Anesthesia, Teikyo University School of Medicine, Tokyo, Japan

**Keywords:** Tracheal bronchus, Anesthesia, One-lung ventilation, Main bronchus angle, Bronchial blocker, Double-lumen tracheal tube

## Abstract

**Background:**

Establishing one-lung ventilation (OLV) in patients with tracheal bronchus (TB) may be challenging due to its unusual bronchial anatomy. We present a case of difficult OLV in a patient with right TB and steeply angled bifurcation of the left main bronchus.

**Case presentation:**

A 79-year-old woman was scheduled to undergo video-assisted thoracic surgery left upper lobectomy. We planned right OLV with a bronchial blocker; however, it was difficult to place the blocker in the left main bronchus due to a steep bifurcation angle. Therefore, we changed the entry angle of the lumen tip by advancing the tracheal tube to just above the tracheal bifurcation, allowing successful placement of the bronchial blocker into the bronchus.

**Conclusion:**

For airway management in patients with TB, especially for OLV, it is essential to understand the anatomy of the trachea, bronchus, and TB and to select the appropriate device for each case.

## Background

Tracheal bronchus (TB) is a rare congenital malformation in which the upper lobe bronchus branches directly from the trachea. Recent reports indicate that the prevalence of true TB in adults, in which the entire right upper lobe bronchus branches directly from the trachea, is less than 1% [1, 2]. TB occurs most commonly in the right lung [1, 2] and usually branches off from the trachea within 2 cm above the tracheal bifurcation [1, 2]. When the TB branches off at a higher level, there are several concerns, such as hypoxia or atelectasis, resulting from obstruction of the TB by an endotracheal tube [3, 4]. We present the case of a patient with right TB, who presented a challenge during bronchial blocker (BB) placement and lung isolation despite preoperative airway evaluation and discussion of airway management. We review the existing literature on TB in thoracic surgery and discuss the strategy of one-lung ventilation (OLV) for patients with TB.

## Case presentation

Institutional review board approval was not required for a single-patient case report at Saitama International Medical Center. Written informed consent was obtained from the patient for this case report. A 79-year-old woman (weight 66.2 kg, height 156 cm) presented to the operating room for video-assisted thoracic surgery left upper lobectomy and resection of anterior mediastinal nodule. Preoperative computed tomography and bronchoscopy revealed right TB branching 2.8 cm above the tracheal bifurcation. The patient had no history of pulmonary surgery and had undergone general anesthesia with endotracheal intubation on two prior occasions without complications. Other preoperative examinations, including blood tests, electrocardiogram, and transthoracic echocardiogram, were unremarkable. OLV with a single-lumen endotracheal tube (SLT) and BB was planned for the procedure.

**Fig. 1:**
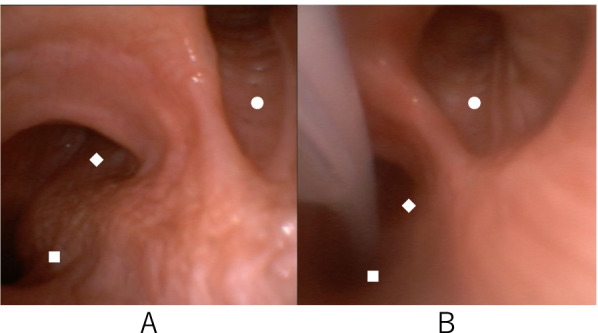
Bronchoscopy before (**A**) and after (**B**) bronchial blocker placement. ■: Left main bronchus ◆: right main bronchus ●: tracheal bronchus

The patient was transported to the operating room, and standard ASA (American Society of Anesthesiologists) monitors were placed. After placing the thoracic epidural catheter, anesthesia was induced with propofol, fentanyl, and rocuronium. The trachea was intubated using an 8.0 mm SLT (TaperGuard™, Covidien, Mansfield, USA), and the tube was secured at the left angle of the mouth. Following anesthesia induction and tracheal intubation, the right TB branching from the trachea was identified using video bronchoscopy (Ambu®aScopeTM 4 Broncho Slim compatible with Ambu® aViewTM, Ambu, Baltorpbakken, Denmark) (Fig. 1 A), and the tip of the SLT was secured just above the branching point. Subsequently, a BB (COOPDECH Endobronchial Blocker Tube; Daiken Medical Co., Ltd., Osaka, Japan) was inserted and advanced to the tracheal bifurcation through the SLT under bronchoscopic guidance. Although the BB was advanced relatively easily to just above the tracheal bifurcation, it was difficult to guide it to the left main bronchus. Therefore, the SLT was advanced just above the bifurcation so that the tip of the BB was directed toward the bronchus, allowing BB placement into the left bronchus (Fig. 1 B). The SLT was then pulled such that its tip was just above the TB branch (19 cm from the lip), and OLV was initiated. It took approximately 30 min from the induction of anesthesia to the start of OLV.

Anesthesia was maintained with desflurane, remifentanil, and an incremental dose of rocuronium, as needed. Conversion to an open chest procedure was required during the operation and completed without complications. Intraoperative respiratory and hemodynamic stability was maintained, and no issues with deflation or ventilation of each lung occurred. At the end of the procedure, the trachea was extubated in the operating room. The duration of anesthesia, surgery, and OLV were 436, 330, and 300 min, respectively. The patient was transported to the intensive care unit and discharged 14 days after surgery. Her postoperative course was uneventful, with no major complications observed.

## Discussion

**Fig. 2:**
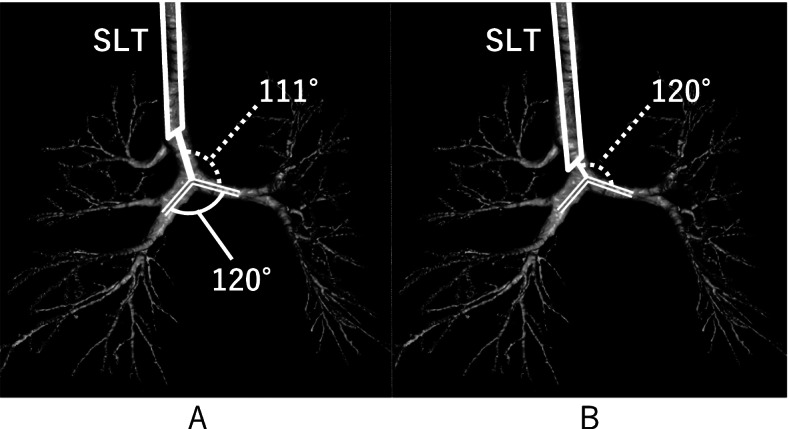
Three-dimensional (3D) image evaluation of the airway. Entry angles from the SLT tip were 111° and 120° when the tip was placed above the TB entry (**A**) and tracheal bifurcation (**B**), respectively. Main bronchus angle was 120° (**A**). SLT: single-lumen endotracheal tube, TB: tracheal bronchus

In patients with TB, OLV can be challenging. To date, no major complications have been reported with OLV in patients with TB; however, as in our case, it can be difficult to place a BB or double-lumen tracheal tube (DLT) into the bronchus. We considered two reasons for the increased time required to place the BB in our case. First, the entry angle from the trachea to the left main bronchus in our case was steeper than usual (111° vs 135°) (Fig. 2 A). Kawagoe et al. reported that the angle of the left main bronchus would become steeper after left upper lobectomy [5], but there was no history of pneumonectomy in this case. Qi et al. reported that the main bronchus angle, defined as the angle between the left and right bronchi, was larger in patients with TB (70.6°−116.5°) than in healthy controls (45.5°−73.6°) [6]. Postoperatively, we reviewed the patient’s preoperative airway CT using a 3D image analysis software (Volume Analyzer, Synapse Vincent, FUJIFILM, Japan) (Fig. 2) and found that the bronchial angle was approximately 120° (Fig. 2 A). Wiser et al. reported failed placement of a DLT for a similar reason [7]. Yoshimura et al. reported successful placement of a left-sided DLT but the failure of OLV due to obstruction of the left bronchial lumen caused by a steep bifurcation angle between the left main bronchus and the trachea [8]. Given these reports, caution should be exercised when planning the insertion of a left-sided DLT in patients with TB. In our case, the left main bronchus was properly occluded despite the initial difficulty in guiding the BB. We overcame this difficulty by inserting the tip of the SLT near the tracheal bifurcation. This lower SLT position directly above the tracheal bifurcation allowed a gentler entry angle into the left main bronchus (120°) compared to the entry angle directly above the TB branch (111°), suggesting that the BB was easier to guide (Fig. 2). If the main bronchus is branched at a steep angle, an SLT with a curved tip (e.g., Parker endotracheal tube, Mercury Medical, Florida) may be advantageous for BB manipulation. However, Parker tubes have a thinner tip than regular tubes, and damage to BB balloons has been reported when they are used with bronchial fibers [9].

Second, the bronchoscope and BB were initially operated by one anesthesiologist, and these interfered with each other and made guiding the BB difficult. Smaller bronchoscopes or BBs with guidewires (Arndt endobronchial blocker, COOK MEDICAL LLC, USA) are recommended to improve the manipulation [4]. Another technique to introduce the BB [(COOPDECH endotracheal blocker tube, DAIKEN MEDICAL, Japan) or (Phycon TCB bronchial blocker, Fuji Systems, Japan)] is using an intravascular guidewire (such as (Radifocus,TERUMO, Japan). In our case, a video bronchoscope was used; thus, information on the airway anatomy could be shared. If anatomical abnormalities are found, it is important to share this information within the team and work to resolve the problem.

**Fig. 3:**
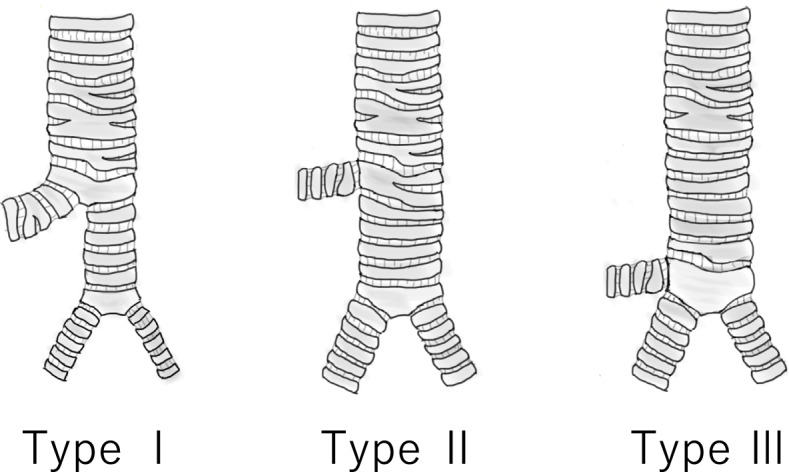
Modified schematic diagram of right TB classification (Conacher’s classification) [9]. Types I and II: the right TB is distant (> 2 cm) from the tracheal bifurcation. The distal trachea is either a narrow bronchial structure (type I) or a normal tracheal structure (type II). Type III: the right TB is close (< 2 cm) to the tracheal bifurcation and appears as a three-branching structure. TB: tracheal bronchus

**Fig. 4:**
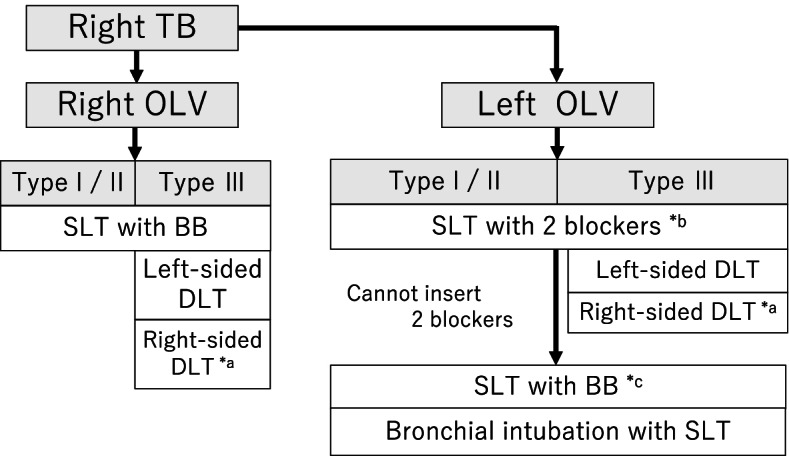
Strategy for OLV in patients with a right TB. *a: Using a right-sided DLT with a shortened bronchial side tube design. *b: Dual BB technique; BB (e.g., Arndt endotracheal blocker or uninvent bronchial blocker) and Fogarty catheters are inserted inside and outside the SLT, respectively, to occlude the right main bronchus and right TB [6, 11]. *c: Selective BB; since the right upper lobe is not deflated by this technique, this technique is dependent on the type of surgical procedure being performed. BB, bronchial blocker; DLT, double-lumen endotracheal tube; OLV, one-lung ventilation; SLT, single-lumen endotracheal tube; TB, tracheal bronchus

Conacher categorized TB into three types based on its distance from the tracheal bifurcation and the tracheal diameter after the TB bifurcation (Conacher’s classification: Fig. 3). This classification is of great significance when considering tracheal intubation and OLV [10]. Use of DLT remains the gold standard technique for various surgical procedures that require lung isolation. However, DLT should be avoided in TB types I and II; especially in type I, DLT is anatomically inapplicable because the diameter of the left-side bronchus at the first branch is usually too small to allow advancement of the DLT into the left-side bronchus at the second branch. Preoperative diagnosis of TB is paramount for anesthetic management. Therefore, it is necessary to identify the airway anatomy including trachea, bronchus, and TB preoperatively, and to select the most appropriate airway-securing device for the surgery [10, 11]. The strategy for establishing OLV for right TB is summarized in Fig. 4.

When right OLV is required, the left bronchus can usually be blocked with a BB passing through a SLT [4]. However, in cases with a larger bifurcation angle of the left main bronchus, as in this case, BB insertion may be difficult [7, 8]. For type III TB, left-sided DLT can be used. As another option for type III TB, where the TB opening is close to the right main bronchus, is a right-sided DLT (Cliny, Create Medic Co., Ltd., Japan) that can be used even in cases with a short right bronchus. In any case, it is necessary to confirm with bronchoscopy that the TB branch is not obstructed by the DLT or SLT.

For left OLV, as a first choice, BB and Fogarty catheters are inserted inside and outside the SLT, respectively, to occlude the right main bronchus and right TB (Dual BB technique) [7, 12]. If the technique is considered difficult, single BB would be the second choice, which is similar to selective BB. Since the right upper lobe cannot be deflated with selective BB, this technique can only be used depending on the surgical procedure. In types I and II, DLT should be avoided because of the possibility of inadequate deflation of the right upper lobe, even if DLT could be inserted. Lai et al. reported that it was possible to deflate the right lobe by adjusting the cuff pressure of the tracheal tube [13]; however, it should be avoided, especially during pneumonectomy, because of the difficulty in managing secretion and bleeding from the right upper lobe. As another option for left OLV, bronchial intubation with SLT in pediatric case is reported [14]. However, the first bifurcating left-side bronchus is narrow, and there is a high possibility that a normal tracheal tube will not be long enough for intubation. Given the concerns, the use of a special tube, such as a long-reinforced tube (Fuji Systems Inc., Japan), may solve the problem. In type III TB, as noted above, the use of DLT is also possible. Kin N et al. reported that a BB could be used for simultaneous occlusion of the right TB and middle bronchus [15]. However, there is a possibility that their case may not have been a true TB, but just a short right main bronchus.

In summary, we present an anecdotal experience with OLV in a patient with right TB. Despite initial difficulties, we were able to properly achieve right OLV by using a BB through a SLT and adjusting the entry angle to access the main bronchus. For airway management in patients with TB, especially for OLV, it is essential to understand the anatomy of the trachea, bronchus, and TB and to select the appropriate device for each case.

## Data Availability

Not applicable.

## Abbreviations

BB: Bronchial blocker
DLT: Double-lumen tracheal tube
OLV: One-lung ventilation
SLT: Single-lumen endotracheal tube
TB: Tracheal bronchus
